# A Large Basal Cell Carcinoma Treated With Hedgehog Inhibitor: A Case Report

**DOI:** 10.7759/cureus.73559

**Published:** 2024-11-12

**Authors:** Ryan Koch, Aaron Chen, Kent Aftergut

**Affiliations:** 1 College of Medicine, Texas A&M College of Medicine, Dallas, USA; 2 Dermatology, Dermatology Associates of Uptown, Cedar Hill, USA

**Keywords:** basal cell carcinoma (bcc), basal cell carcinoma diagnosis, hedgehog pathway inhibitors, locally advanced bcc, vismodegib

## Abstract

Basal cell carcinoma (BCC) is the most commonly diagnosed cutaneous cancer globally. Chronic exposure to environmental triggers and genetic predisposition are risk factors that contribute to the incidence of BCC. While most cases of BCC are treated surgically with curettage or simple excision, treatment options for advanced BCC, including metastatic BCC and locally advanced BCC, are limited as some may be considered unresectable. Advancements in the role of hedgehog signaling in the pathogenesis of BCC have resulted in the development of hedgehog pathway inhibitors as the best treatment for BCC. Germline or somatic mutations in hedgehog pathway signaling components (Smoothened, Patched-1, etc.) result in constant activation of this pathway. Sonidegib and vismodegib are synthetic mimetics of hedgehog pathway inhibitors that are indicated for many subtypes of advanced BCC. We report an unusual case of locally advanced BCC in a 61-year-old male who suffered from a growing BCC for at least six years. After eight months of hedgehog pathway inhibitor (HHI) therapy, the patient reported dramatic improvements in his BCC and complete regression of smaller BCCs previously noted on his upper extremities. The patient reported only minor adverse events including hair thinning, weight loss, and rapidly growing nails.

## Introduction

Basal cell carcinoma (BCC) is the most common type of skin cancer [1]. It typically arises from the basal layer of the epidermis or hair follicle stem cells, and its development is strongly linked to chronic exposure to ultraviolet radiation, particularly ultraviolet B radiation, which causes DNA damage. This DNA damage often leads to mutations in key tumor suppressor genes, such as Patched-1 (PTCH1), which is integral to the hedgehog (HH) signaling pathway. The incidence of BCC continues to rise globally, especially in populations with lighter skin types and increased exposure to sunlight. In the United States, more than four million new cases of BCC are diagnosed each year, making it a significant public health concern. Although BCC is rarely fatal, it can cause substantial morbidity due to its potential for local invasion and tissue destruction, particularly in advanced or neglected cases [1,2].

The majority of BCCs are slow-growing and can be effectively treated with surgical excision or other local treatments, such as curettage, cryotherapy, or photodynamic therapy. Mohs micrographic surgery is widely regarded as the gold standard for high-risk BCCs or those in cosmetically sensitive areas due to its high cure rates and tissue-sparing approach [1]. However, a subset of BCC cases progresses to locally advanced BCC (laBCC) or metastatic BCC (mBCC), which are challenging to manage due to their unresectable nature and resistance to traditional therapies. In these cases, systemic therapies, particularly hedgehog pathway inhibitors (HHIs), have emerged as a promising alternative. The HH signaling pathway is essential for embryonic development and tissue homeostasis, but its aberrant activation, usually through mutations in PTCH1 or Smoothened (SMO) genes, plays a pivotal role in BCC pathogenesis. The discovery of this pathway’s involvement in BCC has led to the development of targeted therapies, such as vismodegib and sonidegib, which specifically inhibit components of the HH pathway and are now approved for use in patients with advanced BCC [3,4]. Despite these advances, many challenges remain, as HHI therapy is not without limitations. Common adverse effects of HHIs include muscle spasms, change in taste, alopecia, weight loss, and fatigue [2,5].

## Case presentation

**Figure 1:**
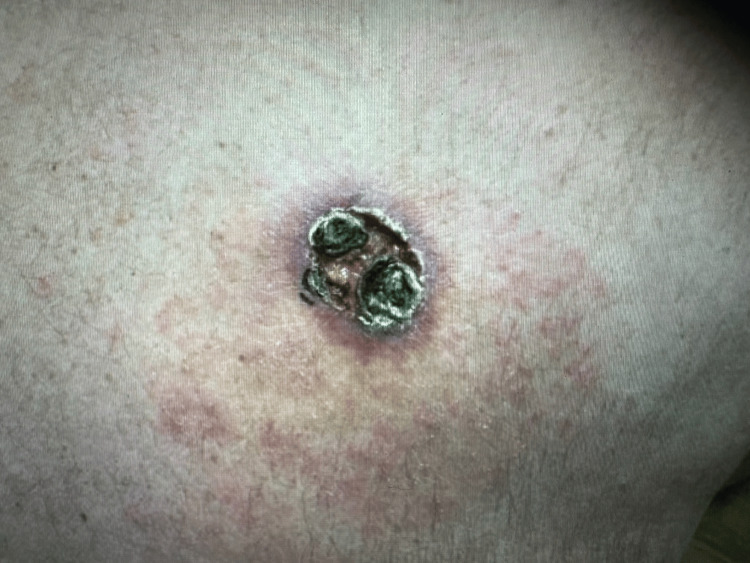
BCC lesion on the patient's upper back at initial visit. BCC: basal cell carcinoma

**Figure 2:**
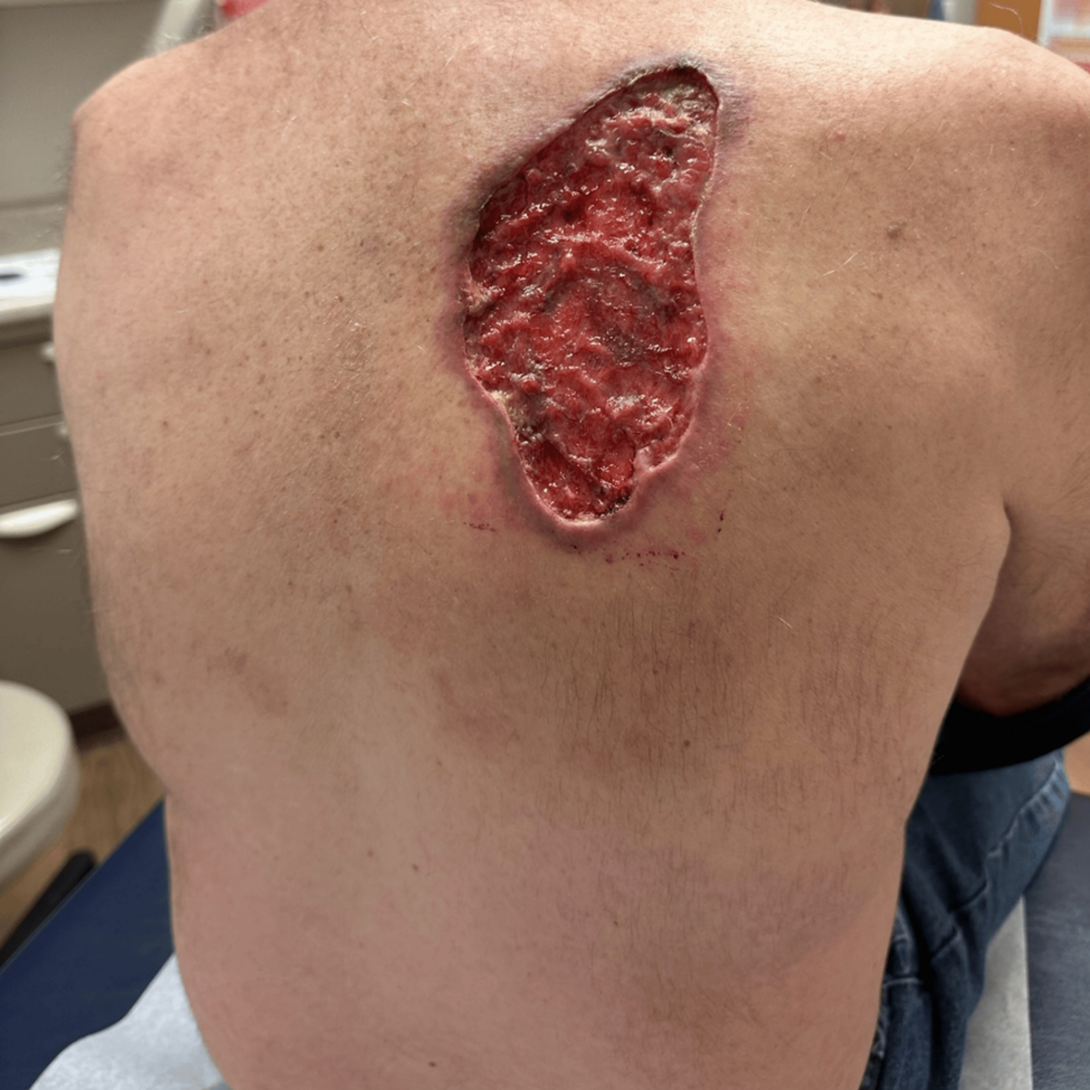
BCC lesion on upper back before starting HHI therapy. BCC: basal cell carcinoma; HHI: hedgehog pathway inhibitor

A 61-year-old male with a past medical history of hypertension initially presented to the dermatology clinic six years ago with a large, biopsy-confirmed nodular BCC on his right upper back (Figure 1). At the time of his initial presentation, surgical options were thoroughly discussed with the patient, including the recommendation for Mohs micrographic surgery. However, the patient elected not to undergo surgery and chose not to return for further treatment against medical advice. His decision not to pursue treatment at that time significantly contributed to the lesion's continued growth over the following years. Five years later, the patient returned to the dermatology clinic after noticing a substantial increase in the size of the BCC, which had now grown to 9 × 5 cm and encompassed a much larger area of his right upper back (Figure 2).

**Figure 3:**
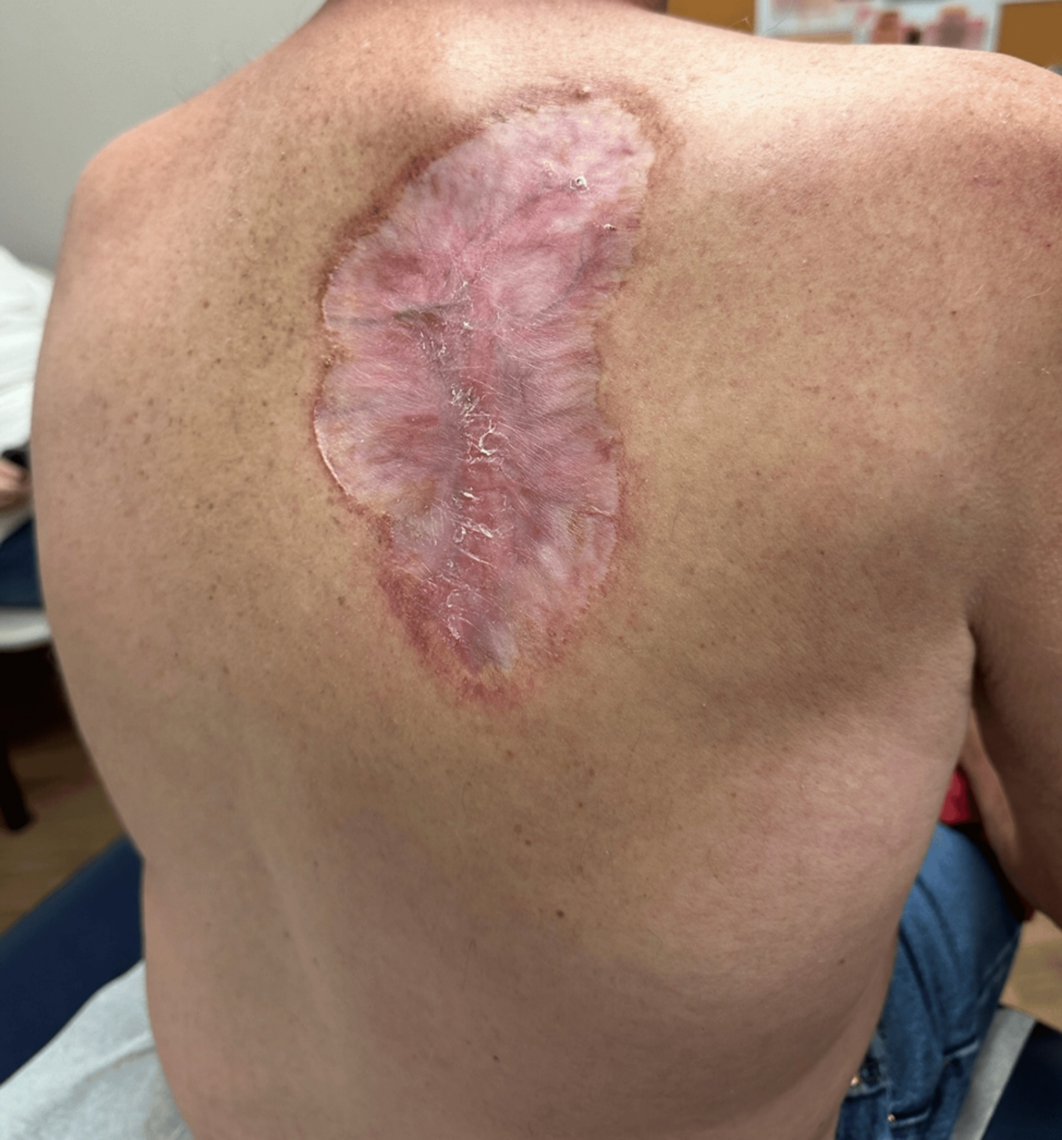
BCC lesion on upper back after eight months of HHI. BCC: basal cell carcinoma; HHI: hedgehog pathway inhibitor

Given that the tumor would be difficult to treat surgically and the patient’s desire to avoid surgery, he was offered an HHI. The patient opted to begin treatment with vismodegib 150 mg by mouth daily, a systemic SMO antagonist approved for treating advanced BCC. After eight months of HHI therapy, the patient experienced significant improvement with a dramatic reduction in the size of the primary BCC lesion on his back (Figure 3). Additionally, smaller BCCs previously noted on his upper extremities completely regressed during this period. The patient tolerated the therapy well, with only minor adverse events reported, including hair thinning, a 20-pound weight loss, and rapid nail growth. He denied experiencing muscle spasms, dysgeusia, or other significant side effects. Interestingly, the patient also reported that he was able to discontinue his antihypertensive medication after the weight loss associated with the therapy. It was recommended that the patient continue HHI therapy for an additional eight weeks with close monitoring for any potential adverse effects. Because of the large size of the original lesion and to avoid multiple scouting biopsies, the lesion will be followed clinically.

## Discussion

The HH signaling pathway plays a crucial role in regulating cell growth, development, and tissue homeostasis. Its activation is tightly controlled under normal physiological conditions, ensuring that cellular proliferation and differentiation occur only as needed for repair and regeneration. The HH signaling pathway is activated via Sonic hedgehog (SHH), Indian hedgehog, or Desert hedgehog to Patched-1 (PTCH1). The canonical HH pathway proceeds through the developmental signaling cascade. Signaling is initiated when there is insufficient ligand binding, resulting in PTCH1-mediated inhibition of Smoothened (SMO), a transmembrane protein that activates the HH signaling cascade [3]. Promotion of the HH signaling pathway results in the activation of glioma-associated oncogene homolog 1 (Gli1), a transcriptional effector that activates cyclin-D1, Myc, and Bcl-2. Non-canonical HH signaling can occur via mechanisms external to HH signaling, including K-ras, transforming growth factor β (TGF-β), and phosphoinositide-3-kinase (PI3K). In many cases, BCC is associated with the upregulation of the HH signaling pathway in the intrafollicular epidermis and progenitor cells of the hair follicles. Furthermore, over 80% of patients with BCC have a loss-of-function mutation in one PTCH1 allele, while the remaining have gain-of-function mutations in one SMO allele [3].

The identification of the hedgehog pathway as a key player in BCC pathogenesis has significantly advanced the therapeutic landscape, particularly for advanced cases that are deemed unresectable. Prior to the development of HHIs, options for managing laBCC or mBCC were limited and often ineffective. The introduction of HHIs like vismodegib and sonidegib has provided a targeted approach to disrupt this pathway, offering patients with advanced BCC a viable non-surgical treatment option [4].

HHIs work by binding to and inhibiting SMO, preventing the downstream activation of the HH signaling cascade. This effectively halts the transcription of genes associated with cell survival and proliferation, such as Gli1, cyclin-D1, Myc, and Bcl-2. As a result, tumor growth is suppressed, and in some cases, tumors can regress entirely. Cyclopamine was among the first compounds that disrupted SMO, resulting in decreased tumor proliferation and progression [3,4]. The adverse effects (AE) of cyclopamine, which included death and teratogenic effects, led to the development of synthetic mimetics. Currently, sonidegib and vismodegib are two systemic SMO antagonists, with sonidegib approved for adult patients with laBCC and vismodegib approved for adult patients with laBCC or mBCC [2]. In this case, vismodegib was successful in reducing the size of the patient’s large BCC and resulted in the complete regression of smaller lesions on his upper extremities. This underscores the potency of HHI therapy in controlling advanced disease, even in cases where the BCC had progressed for several years without intervention.

Despite their efficacy, HHIs are associated with a range of adverse events, many of which can impact patient quality of life and adherence to therapy. The most commonly reported AEs of sonidegib and vismodegib include muscle spasms, alopecia, weight loss, and dysgeusia [2,3]. These side effects are believed to result from the HH pathway’s role in normal physiological processes, such as hair follicle development and muscle function [2].

Prolonged use of HHIs is associated with an increased likelihood of adverse events, which can lead to treatment discontinuation in some patients [6]. To mitigate side effects over extended therapy, pulsed or intermittent dosing has been explored, offering potential relief from side effects while maintaining efficacy [6]. Discontinuation of treatment may increase the risk of tumor recurrence, underscoring the need for careful monitoring. Additionally, the high cost of HHI therapy remains a significant limitation, particularly given the potential need for long-term or recurring treatments.

Administration of L-carnitine, a non-essential amino acid responsible for mitochondrial ATP generation by increasing the β-oxidation of long-chain fatty acids, can stabilize the sarcolemma, permitting muscle relaxation [3]. The HH signaling pathway is necessary for normal hair growth, resulting in HHI-induced alopecia. Co-treatment with HHI and oral finasteride or topical minoxidil is indicated for HHI-induced alopecia. While the mechanism of dysgeusia and HHI therapy is unclear, zinc deficiency is hypothesized to play a role in taste bud function, size, and integrity. Further inquiries are required to understand the benefits of zinc supplementation for patients on HHI therapy [3].

In this case, the patient experienced minor AEs, including hair thinning, weight loss, and rapid nail growth, but did not report the more debilitating AEs that can occur with HHI therapy, such as muscle cramps or severe dysgeusia. The patient’s weight loss, while significant, was not associated with loss of appetite or fatigue and appeared to contribute positively to his ability to discontinue antihypertensive medication. This suggests that individual responses to HHI therapy can vary and that not all patients will experience major side effects.

## Conclusions

Hedgehog pathway inhibitors remain an effective treatment option for patients with advanced BCC, especially those who are not candidates for surgery. This case illustrates the significant efficacy of vismodegib in inducing tumor regression, even in long-standing and untreated BCC. Although adverse events are common, they may be manageable with supportive care and do not always necessitate discontinuation of therapy. Continued research into strategies for minimizing adverse effects and optimizing patient adherence to HHI therapy will be critical as these agents continue to play a role in the management of BCC.
